# Metagenomic Insight into Cecal Microbiota Shifts in Broiler Chicks Following *Eimeria* spp. Vaccination

**DOI:** 10.3390/microorganisms13071470

**Published:** 2025-06-24

**Authors:** Dimitrios Marinos Karadedos, Tilemachos Mantzios, Despoina Eugenia Kiousi, Margaritis Tsifintaris, Ilias Giannenas, Panagiotis Sakkas, Georgios A. Papadopoulos, Gunther Antonissen, Aglaia Pappa, Alex Galanis, Vasilios Tsiouris

**Affiliations:** 1 Department of Molecular Biology and Genetics, Faculty of Health Sciences, Democritus University of Thrace, 68100 Alexandroupolis, Greece; dimikara91@mbg.duth.gr (D.M.K.); mantzios@vet.auth.gr (T.M.); dkiousi@mbg.duth.gr (D.E.K.); mtsifintaris@gmail.com (M.T.); apappa@mbg.duth.gr (A.P.); agalanis@mbg.duth.gr (A.G.); 2 Unit of Avian Medicine, Clinic of Farm Animals, School of Veterinary Medicine, Aristotle University of Thessaloniki, 54627 Thessaloniki, Greece; 3 Laboratory of Nutrition, School of Veterinary Medicine, Aristotle University of Thessaloniki, 54124 Thessaloniki, Greece; igiannenas@vet.auth.gr (I.G.); psakk@vet.auth.gr (P.S.); 4 Laboratory of Animal Husbandry, Faculty of Veterinary Medicine, Aristotle University of Thessaloniki, 54124 Thessaloniki, Greece; geopaps@vet.auth.gr; 5 Department of Pathobiology, Pharmacology and Zoological Medicine, Faculty of Veterinary Medicine, Ghent University, 9820 Merelbeke-Melle, Belgium; Gunther.antonissen@ugent.be

**Keywords:** coccidiosis, broilers, gut health, performance, microbiota, metagenomics, 16S rRNA gene amplicon sequencing

## Abstract

Coccidiosis, caused by *Eimeria* spp., remains a major challenge in poultry production, significantly affecting poultry health and performance, leading to substantial economic losses. While its impact on gut health is well documented, the interplay of *Eimeria* spp. challenge and/or vaccination with the intestinal microbiota remain insufficiently understood. Therefore, the aim of this study was to investigate the effects of *Eimeria* spp. (*E. acervulina*, *E. maxima*, and *E. tenella*) challenge, alone or in combination with a commercially available vaccine, on broiler performance, intestinal gross lesions, and cecal microbiota structure and function in experimentally challenged broiler chicks. A total of 216 Ross 308^®^ broilers were randomly divided into three groups, with six replicates per group, according to the following experimental design: (A) negative control, (B) *Eimeria* spp.-challenged birds on day 16, and (C) *Eimeria* spp.-vaccinated and -challenged birds. Performance parameters were recorded on a weekly basis, coccidiosis gross lesions in the intestine were evaluated on days 23 and 29, and microbiota samples were collected on day 23. Broilers in the challenged group exhibited significantly (*p* ≤ 0.05) increased coccidiosis gross lesions in the intestine at both sampling periods (7 and 19 days post-infection, dpi), whereas vaccination significantly (*p* ≤ 0.05) minimized the severity of lesions at both time points. The challenged-only group showed significantly (*p* ≤ 0.05) lower average daily weight gain (ADWG) during the finisher phase and the overall experimental period compared to the vaccinated group. Additionally, average daily feed intake (ADFI) during the post-challenge period (22–29 dpi) was significantly (*p* ≤ 0.05) reduced in both challenged groups. Alpha diversity decreased in the challenged (*p* = 0.016) and vaccinated–challenged (*p* = 0.016) groups compared to control, Accordingly, beta diversity was reduced in groups B and C compared to the control group. This reduction was accompanied by an increased relative abundance of Proteobacteria (18, 71% in Group B and 10, 87% in Group C) and potentially pathogenic genera (*Escherichia* spp. and *Shigella* spp. *p* < 0.05), along with a decline in short-chain fatty acid (SCFA)-producing bacteria (*Oscillibacter* spp. and *Eisenbergiella* spp.) in groups B and C, respectively, compared to the control. Predictive functional metagenomics indicated disruptions in amino acid metabolism, nucleotide degradation, and lipid metabolism, potentially affecting gut integrity and nutrient absorption. Additionally, in the vaccinated group, gross lesions in the intestine were reduced in severity and microbial diversity was partially preserved, resulting in a microbiota composition more similar to that of the control group. Overall, these findings support that *Eimeria* spp. infection alters gut microbiota and function in broiler chicks, underscoring the need for further research into alternative strategies, such as probiotics and phytobiotics, to support gut health and disease resilience in poultry.

## 1. Introduction

Coccidiosis, caused by protozoan parasites of the genus *Eimeria*, represents a major challenge in the poultry industry, leading to significant economic losses globally [1,2]. Of the seven *Eimeria* species known to infect chickens, *E. acervulina*, *E. tenella*, and *E. maxima* are the most prevalent in commercial broiler systems [3,4]. The disease primarily affects the intestinal tract, where the parasites invade and multiply within epithelial cells, leading to tissue damage, impaired nutrient absorption, disruptions in the gut microbiota, often resulting in intestinal dysbiosis [5,6,7]. These pathological changes result in poor feed efficiency, reduced weight gain, and increased mortality [8]. The severity of infections varies depending on the specific *Eimeria* species involved, with some causing a more significant impact due to higher morbidity and mortality rates [9,10]. Under field conditions, infections are often caused by multiple *Eimeria* species simultaneously, compounding the overall impact on broiler health [11].

While anticoccidial drugs have historically been effective in controlling the disease, their prolonged and widespread use has led to the emergence of drug-resistant *Eimeria* spp. strains [12]. Increasing resistance of *Eimeria* spp. field isolates, particularly *E. tenella*, *E. acervulina*, and *E. necatrix* (as most frequently isolated), has been reported in the majority of commonly used anticoccidial drugs [13,14,15]. These include both ionophore compounds (e.g., salinomycin, monensin, and lasalocid) and synthetic chemicals (e.g., sulfachloropyrazine sodium, amprolium, toltrazuril, clopidol, and nicarbazine) [12]. Growing concerns about sustainability and production, along with increasing regulatory restrictions on the usage of antimicrobial drugs, highlight the need for sustainable alternatives [8,12,16,17,18]. In this context, vaccination has gained increasing traction as a key strategy for coccidiosis control. Live or attenuated *Eimeria* spp. vaccines offer effective protection without promoting resistance in field strains and may even help restore sensitivity to previously ineffective anticoccidials [19,20,21,22,23]. However, despite their use in commercial poultry production, data on how these vaccines influence gut health and microbiota are scare [23,24,25,26]. It is reported that during the early growth phase (days 1–21), the replication of live oocysts from the vaccine within the host can induce mild subclinical coccidiosis, which is associated with reduced intestinal absorptive surface, impaired nutrient absorption, and localized inflammation [25].

The gastrointestinal tract of broilers harbors a complex and diverse microbiota, which plays a pivotal role in maintaining gut health, nutrient absorption, and immune function [27,28]. Among the key microbial groups, members of the *Firmicutes* and *Bacteroides* phyla, along with species such as *Faecalibacterium* spp. and *Butyricicoccus* spp., contribute significantly to the production of short-chain fatty acids (SCFAs), including butyrate [29,30]. These SCFAs not only serve as a major energy source for intestinal cells but also help regulate inflammation and maintain the integrity of the gut barrier [31].

Infections with *Eimeria* spp. are known to induce intestinal inflammation, compromise epithelial integrity, and disrupt microbial homeostasis, often resulting in dysbiosis [23,24,32]. Recent studies have highlighted that *Eimeria* spp. colonization in broilers significantly alters the gut microbiota, including dominant phyla such as *Firmicutes*, *Bacteroidetes*, and *Proteobacteria*. For instance, Qiao et al. (2024) [33] reported that *E. tenella* infection led to pronounced shifts in the cecal microbiota, notably increasing the abundance of *Eubacterium coprostanoligenes*, *Erysipelatoclostridium*, *Shuttleworthia*, and *Colidextribacter* in vaccinated chickens [33]. Similarly, Chen et al. (2020) [34] observed reductions in beneficial taxa such as *Lactobacillus*, *Faecalibacterium*, *Ruminococcaceae* UCG-013, and *Romboutsia* during *E. tenella* infection [34].

Recent studies employing 16S rRNA gene sequencing have demonstrated that anticoccidial vaccination, particularly when combined with dietary interventions (e.g., *Bacillus subtilis*, *Clostridium butyricum*, or berry pomace) can influence microbial community composition, with a reduction in gut microbiome diversity and stability in the first 8 days post-vaccination, followed by partial recovery by day 15 [23,24,25,26]. In a recent multi-omics study, Liu et al. (2024) [35] investigated the effects of a *Saccharomyces cerevisiae*-vectored anticoccidial vaccine in broilers challenged with *E. tenella*. Their findings revealed significant shifts in both the gut microbiota and metabolome. Vaccinated chickens showed an increased abundance of beneficial taxa such as *Bifidobacterium* and *Lactobacillus*, as well as an enrichment in anti-inflammatory sphingolipids, indicating potential immunomodulatory effects [35]. However, systematic data specifically assessing the effects of commercially available *Eimeria* vaccines alone—without concurrent probiotics or supplements—on gut microbiota composition, diversity and functional dynamics remain limited. Moreover, previous studies primarily relied on short-read sequencing platforms [5,9,34], with limited investigation into the functional roles and metabolic potential of the altered microbiota.

In addition, the majority of the research studies [34,36,37] employed high infection doses that induce severe pathological changes [33], conditions that may not accurately reflect the more common subclinical infections observed under field settings [38,39]. Moreover, most studies have focused on single-species *Eimeria* infections, whereas for in-field conditions, mixed-species infections are the norm [38,39], presenting a more complex interaction with the gut microbiota. These gaps underline the need for studies simulating realistic in-field scenarios, incorporating multi-species *Eimeria* challenge and metagenomic approaches to assess microbiota dynamics under subclinical conditions.

Thus, the objective of this study was to investigate the effects of *Eimeria* spp. (*E. acervulina*, *E. maxima*, and *E. tenella*) challenge, alone or in combination with a commercially available vaccine, on broiler performance, intestinal gross lesions, and the cecal microbiome. Hence, we employed full-length 16S rRNA gene sequencing using Oxford Nanopore technology, followed by predictive functional metagenomics, to access changes in both the structural composition and metabolic pathways in experimentally challenged broiler chicks. To our knowledge, this study represents the first integrated structural and functional analysis of the cecal microbiota following *Eimeria* spp. infection using full-length 16S rRNA gene sequencing and predictive functional metagenomics.

## 2. Materials and Methods

### 2.1. Experimental Facilities, Biosecurity, and Ethics

The study was conducted at the experimental facilities of the Unit of Avian Medicine, School of Veterinary Medicine (EL-54-BIOexp-03), Aristotle University of Thessaloniki (AUTh), Greece. All procedures followed the guidelines of the Council Directive (2010/63/EU) and Greek legislation concerning the welfare, husbandry, euthanasia, and biosecurity measures for experimental animals. Ethical approval for the study was obtained from the Ethical Committee of the School of Veterinary Medicine and the Greek Veterinary Authority (420364/1913).

### 2.2. Experimental Design

A total of 216 one-day-old Ross 308^®^ broiler chicks were randomly allocated into three (3) treatment groups, consisting of six (6) replicates/subgroups (12 chicks per pen), according to the following experimental design: group A:unchallenged birds (negative control group), group B: birds were challenged with a mixed inoculum of *Eimeria* spp., and group C: birds were vaccinated with an anticoccidial vaccine (EVANT^®^, HIPRA, S.A., Girona, Spain) on the first day, according to the manufacturer’s recommended dosage (0.007 mL/bird *peros*), and challenged with a mixed inoculum of *Eimeria* spp.

The chicks were randomly assigned to the experimental groups and placed in cages in separate experimental rooms. The cages were metal battery-type and were divided into equal compartments (pens) of 110 cm in width by 50 cm in length. Experimental subgroups were randomly distributed among the experimental rooms to minimize room effects, with only the negative control group housed in a separate room to avoid cross-contamination. Environmental parameters, including temperature, relative humidity, and lighting, were regulated equally across the experimental rooms and monitored daily using a temperature–humidity data logger (HOBO UX100-003, Onset Computer Corporation, Bourne, MA, USA) and adjusted according to the recommendations provided by the breeding company (Aviagen^®^, Huntsville, AL, USA).

A comprehensive biosecurity protocol was implemented to control the movement of equipment, personnel, and feed between the rooms. This protocol aimed to prevent cross-contamination and included the use of dedicated protective clothing, disinfection footbaths at entry points, and separate handling equipment for each group.

### 2.3. Feed

Feed and water were provided *ad libitum* throughout the experiment. To meet the nutrient requirements of growing chicks, two complete basal diets were formulated for the starter (1 to 10 days) and the finishing period (10 to 35 days), respectively, following standard nutritional recommendations of the breeding company (Aviagen^®^, Huntsville, AL, USA). No antibiotic growth promoters, organic acids, essential oils, or related anticoccidial drugs or/and mycotoxin binders were used. The feed synthesis and chemical analysis are shown in Table S1.

### 2.4. Challenge Protocol

The birds in groups B and C were challenged individually on day 16 via esophageal catheter with 20-fold dose of the multi-species *Eimeria* vaccine (EVANT^®^, HIPRA, S.A., Girona, Spain) in order to induce subclinical coccidiosis. The recommended vaccine dose (0.007 mL) contained the following species of *Eimeria*, derived from precocious attenuated lines according to the manufacturer’s in vitro procedures: *E. acervulina*, strain 003 (332–450 sporulated oocysts), *E. maxima*, strain 013 (196–265 sporulated oocysts), *E. mitis*, strain 006 (293–397 sporulated oocysts), *E. praecox*, strain 007 (293–397 sporulated oocysts), and *E. tenella*, strain 004 (276–374 sporulated oocysts). Therefore, in the present study, the 20-fold dose of the *Eimeria* spp. vaccine used for the challenge of birds resulted in the following estimated oocyst loads per species: *E. acervulina*, strain 003: 6640–9000, *E. maxima*, strain 013: 3920–5300, *E. mitis*, strain 006: 5860–7940, *E. praecox*, strain 007: 5860–7940, and *E. tenella*, strain 004: 5520–7480 sporulated oocysts.

### 2.5. Performance Evaluation

During this trial, clinical signs and/or mortality were recorded daily. To evaluate the effect of the *Eimeria* spp. challenge and the anticoccidial vaccination on the performance of broilers, the body weight (BW) of the birds was individually measured on 1st, 9th, 16th, 20th, 22nd, 24th, 29th, and 35th day of age, while the average daily feed intake (ADFI), the average daily weight gain (ADWG), and the feed conversion ratio (FCR) were calculated at pen level for the periods 1st–9th, 9th–22nd, 22nd–29th, 29th–35th, and 1st–35th days.

### 2.6. Gross Lesion Scoring (LS)

Two samplings were conducted on days 23 (7 dpi) and 29 (13 dpi). Prior to necropsy, broiler chicks were euthanized by exposure to a rising concentration of carbon dioxide (CO_2_) in an air-tight container, following all institutional and national guidelines for the handling of laboratory animals, ensuring ethical and responsible practices throughout the research process.

#### 2.6.1. Coccidiosis LS

The duodenum, jejunum, ileum, and caeca from each bird in all samplings (23rd and 29th day) were collected, macroscopically examined, and scored for coccidial gross lesions. In particular, the intestines were macroscopically examined and scored on a scale ranging from 0 to 4 for coccidiosis lesions (Jonhson and Reid, 1970), where 0 reflects a normal gastrointestinal tract and 4 reflecting the most severe gross lesions, characteristic for each *Eimeria* species [40,41]. The coccidiosis score was evaluated for each *Eimeria* strain: *E. acervulina* in the duodenum, *E. maxima* in the duodenum/jejunum, and *E. tenella* in the caeca. The total mean lesion score (TMLS) for coccidiosis was calculated as the sum of the average of the individual mean lesion scores for *E. acervulina* and *E. tenella* [40,41].

#### 2.6.2. Dysbiosis LS

Intestines were macroscopically examined and scored for dysbiosis lesions on day 27 (13 dpi) as previously described by Mantzios et al. [42]. Each bird was given a score between 0 and 10, where 0 reflects a normal gastrointestinal tract and 10 if the most severe dysbiosis lesions occurred. For this reason, a total of 6 parameters [ballooning (0–1), inflammation (0–2), thickness (0–2), content characteristics (0–2), flaccid (0–2), and undigested feed (0–1)] were assessed, and finally, individual scores were summed to obtain the final dysbiosis score for the intestine of each bird.

### 2.7. Harvesting of Caeca

Following necropsy, on day 23 (7 dpi), caeca from 2 birds/subgroup (12 birds/group) were aseptically collected and pooled by group in sterile stomacher bags. The mixture was homogenized for 10 min using a Stomacher (Interscience, Saint Nom la Bretêche, France), and the samples were stored at −80 °C, until further analysis.

### 2.8. DNA Extraction and 16S rRNA Oxford Nanopore Sequencing

One pooled sample per group was collected. Each pool consisted of cecal material from six (6) replicates/subgroups (12 chicks per group) to capture group-level variability while minimizing within-group variability. Total DNA extraction was performed on five technical replicates per pooled sample using the MagCore^®^ Gut Microbiome DNA Kit (Code: 504) on the MagCore^®^ Plus II Automated Nucleic Acid Extractor (RBC Bioscience, New Taipei City, Taiwan). The quality and quantity of the extracted DNA were determined by NanoDrop^®^ ND-1000 UV–Vis (Thermo Fisher Scientific, Waltham, MA, USA). DNA samples were stored at −20 °C. The extracted DNA was sequenced using the Oxford Nanopore MinION^®^ (Oxford Nanopore Technologies, Oxford, UK). It should be noted that prior to sequencing, DNA from each sample was quantified and normalized, so equimolar concentration of DNA was used for library preparation and sequencing for each sample. Libraries were prepared using the 16S Barcoding Kit 24 V14 (Oxford Nanopore Technologies). Read characteristics such as quality, length, and base yield above length were calculated using the 16S workflow in the free access Oxford Nanopore software EPI2ME Labs software v5.2.0 (Oxford Nanopore Technologies) “https://nanoporetech.com/products/analyse/epi2me/” (accessed on 23 November 2024) desktop client. Demultiplexed read data were exported in FASTQ format and used as input to the free access ONT-AmpSeq pipeline (v1.1.1) developed by Schacksen et al. [43] for further analysis.

First, chopper [44] was used to filter out raw reads smaller than 1000 bp and larger than 1800 bp, as well as reads with a quality score lower than 13. Vsearch v2.29.4 [45] was implemented to cluster reads into Operational Taxonomic Units (OTUs) and denoised using UNOISE3 [46]. OTUs polishing was performed by Racon [47] to minimize sequencing errors. Taxonomy annotation of the OTUs was performed using the BLAST v2.16.0 algorithm [48], into NCBI’s bacterial 16S rDNA database [49], using the default e-value threshold (1 × 10^−10^).

Alpha (Chao1, Shimpson’s, Shannon and Pielou’s evenness) and beta diversity (Bray–Curtis and Jaccard dissimilarity) indices were calculated using the phyloseq R package v1.48.0 [50]. Alpha diversity indices were calculated on raw count data. Pairwise comparisons of alpha diversity between treatment groups were performed using the Wilcoxon rank-sum test, for beta diversity reads were normalized by rarefaction. Principal Coordinates Analysis (PCoA) was performed to visualize beta diversity patterns and assess microbial community differences among experimental groups. Beta diversity differences were assessed using PERMANOVA (Permutational Multivariate Analysis of Variance) with multiple testing correction, as implemented in the vegan R package v. 2.9-10 [51]. Relative abundance bar plots and Venn diagrams demonstrating the similarity between microbial communities across studied groups were generated using ggplot2 v3.5.1 [52]. Differential abundance analysis of the data was performed using LEfSe v1.1.2 (Linear discriminant analysis effect size) [53] and *p*-values were corrected for multiple testing using the Benjamini–Hochberg false discovery rate (FDR) method. Furthermore, picrust2 v2.4.2 (Phylogenetic Investigation of Communities by Reconstruction of Unobserved States) [54] was implemented to perform functional analysis based on marker genes with default settings and the results were visualized using ggpicrust2 v1.7.4 [55]. Pathways with log_2_ fold change ≥ 1 and *p* ≤ 0.05 were considered differentially abundant.

### 2.9. Statistical Analysis

The effect of *Eimeria* spp. challenge, alone or in combination with a commercial *Eimeria* spp. vaccine, on broiler performance parameters was analyzed using one-way ANOVA in SPSS 28.0 (IBM SPSS Statistics for Windows, Version 28.0, Armonk, NY, USA: IBM Corp.). Post hoc comparisons between treatments were conducted using Duncan’s and Tukey’s tests. Mean values and their standard deviations were calculated for all examined parameters (BW, ADFI, ADWG, and FCR). Lesion score analysis was performed using both SPSS and GraphPad Prism (Version 9.1.2 for Windows^®^, GraphPad Software, San Diego, CA, USA). Specifically, for parameters evaluated as scores (coccidiosis lesion score in the intestine), cross-tabulation and Khi-2 analysis were conducted in SPSS, followed by Kruskal–Wallis tests [56], which were performed either in SPSS or GraphPad Prism. The level of significance was set at *p* ≤ 0.05.

## 3. Results

### 3.1. Performance Evaluation

Throughout the entire period (1–35 days), mortality was not recorded. Daily clinical examination revealed sporadic clinical signs in the challenged groups, such as diarrhea, ruffled feathers, and growth retardation. The effects of the *Eimeria* spp. challenge and its combination with anticoccidial vaccination on the performance parameters of broiler chicks are presented in Table 1. Neither the challenge alone nor in combination with vaccination significantly (*p* > 0.05) affected the BW of the birds at any of the measured time points (Table 1).

The ADWG in broilers during the finisher (22–35 days) and total experimental period (1–35 days) was significantly (*p* ≤ 0.05) lower in group B compared to group C. Additionally, the ADFI during the post-challenge period (22–29 days) was significantly (*p* ≤ 0.05) higher in groups B and C compared to group A. Finally, no significant differences were observed in the FCR. Nevertheless, while some differences were statistically significant, their biological relevance appeared modest.

### 3.2. Gross Lesion Scoring (LS)

#### 3.2.1. Coccidiosis LS

The effects of the *Eimeria* spp. challenge and its combination with anticoccidial vaccination on coccidiosis lesion scores in the intestines of broiler chicks are presented in Table 2. No gross coccidiosis lesions were observed in the intestines of birds in experimental group A, underscoring the effectiveness of the strict biosecurity measures implemented during the study. In group B, mild *E. acervulina* lesions were detected in 27.8% of the intestines during the 1st sampling (22.2% with a score of 1 and 5.6% with a score of 2), which was significantly higher compared to group A (*p* = 0.008; 100% with a score of 0) and group C (*p* = 0.034; 5.6% with a score of 1). During the second sampling, *E. acervulina* lesions were observed in 5.9% of the intestines in group B and 5.6% in group C, all presenting a lesion score of 1. For *E. maxima*, no lesions were recorded in any group during either sampling.

*E. tenella* lesions were observed in a significant (*p* < 0.001) proportion of birds in group B during both samplings. In the first sampling, lesions were present in 100% of the birds in group B (*p* < 0.001), with 16.7% scoring 1 and 83.3% scoring 2. In contrast, in group C, *E. tenella* lesions were detected in 55.6% of the intestines (*p* < 0.001), with 38.9% scoring 1 and 16.7% scoring 2. During the second sampling, *E. tenella* lesions (*p* < 0.001) were recorded in 76.5% of the birds in group B, with 58.9% scoring 1 and 17.6% scoring 2. In group C, 33.3% of the intestines had lesions, all scoring 1.

#### 3.2.2. Dysbiosis LS

The effects of the *Eimeria* spp. challenge and its combination with anticoccidial vaccination on coccidiosis lesion scores in the intestines of broiler chicks are presented in Table 3. The intestines of broilers in the experimental groups B and C exhibited significantly (*p* = 0.05) more dysbiosis lesions compared to the negative control group (group A).

### 3.3. 16S rDNA Sequencing Read Characteristics

Amplicon metagenomic sequencing generated a total of 422,820 reads (post-filtration); 223,829 reads were assigned to group A (control group), 97,816 to group B (challenged group), and 101,175 to group C. During quality control, reads with quality lower than 13 and with lengths shorter than 1000 bp and 1800 bp were excluded from downstream analysis. The mean and median length of the reads in each sample ranged from 1400 to 1500 bp and the N50 value was 1000 bp for each sample. The mean read quality score for all samples was approximately 15. Sequencing depth per sample ranged from 10,492 to 60,536 reads, with a mean of 32,602 and a median of 33,715 reads per sample. (Table S2). Of the reads generated, 90.9% were assigned to Operational Taxonomic Units (OTUs) at kingdom, phylum, class, order, family, genus, and species taxonomic ranks. The remaining 9.1% were not classified into a taxonomic rank (unclassified OTUs). A total of 90.25% of the classified reads were assigned to (OTUs) at the genus level, while 90.2% were classified at the species level.

### 3.4. Bacterial Diversity

**Figure 1:**
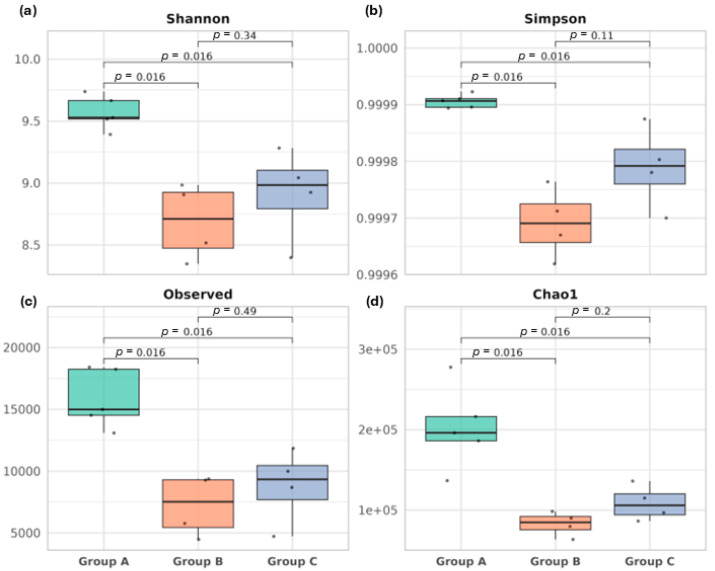
Boxplots show the distribution of alpha diversity calculated by (**a**) Shannon diversity index, (**b**) Simpson diversity index, (**c**) observed species (richness), and (**d**) Chao1-estimated richness for each group; group A (control, in green), group B (challenged with *Eimeria* spp., red), and group C (vaccinated and challenged with *Eimeria* spp., in blue). Horizontal bars indicate pairwise comparison between groups, with the corresponding *p*-values from Wilcoxon rank-sum tests shown above. Significant differences (*p* < 0.05) are observed between the control and challenged groups for all indices, indicating a reduction in microbial diversity and richness following *Eimeria* spp. challenge. Each dot represents a pooled sample.

Bacterial diversity for each group was determined by measuring richness and evenness of the microbial communities within a group (alpha diversity). Four indices were calculated to determine alpha diversity across samples, the Shannon–Wiener index, Simpsons’ index, Chao1 index, and Pielou’s Evenness (Figure 1). Specifically, significant differences in alpha diversity were recorded between the control and challenged groups; however, no difference was reported for groups B and C.

The higher Shannon index value in the control group indicates a more stable microbial community, characterized by even distribution of taxa compared to groups B and C. Similarly, the Simpson index value is highest in group A, indicating greater species diversity and a more even distribution of taxa compared to groups B and C. The Chao1 index, which estimates species richness, was highest in group A, followed by group C, while group B exhibited the lowest species richness. Pielou’s evenness values were also highest in group A, suggesting a more even distribution of bacterial taxa, whereas groups B and C displayed lower evenness, indicating greater dominance of certain taxa (Figure 1, Table S3).

Overall, these findings demonstrate that group A harbors the highest alpha diversity, characterized by greater species richness and a more even taxonomic distribution. In contrast, group B exhibits reduced alpha diversity, while group C shows intermediate diversity levels, with richness and evenness values falling between those of groups A and B.

**Figure 2:**
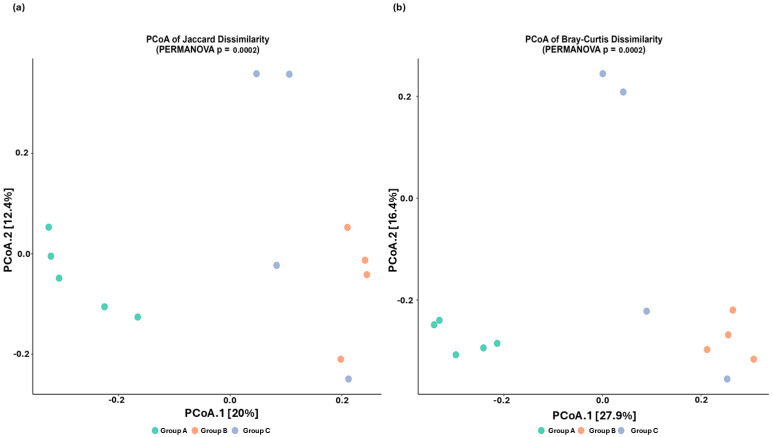
Principal Coordinates Analysis (PCoA) of cecal microbiota beta diversity across experimental groups. (**a**) PCoA plot based on Jaccard dissimilarity, with PCoA 1 and PCoA 2 explaining 20% and 12.4% of the variation in Operational Taxonomic Units (OTUs), respectively. (**b**) PCoA plot based on Bray–Curtis similarity, with PCoA 1 and PCoA 2 explaining 27.9% and 16.4% of the variation in OTUs, respectively. Each dot represents a pooled sample from each experimental group (green, group A; red, group B; and blue, Group C). PERMANOVA analysis indicates statistically significant differences in microbial community composition among groups, (*p* = 0.0002).

PCoA was performed to investigate beta diversity. Bray–Curtis dissimilarity and Jaccard indices were used to evaluate differences in microbial community composition among groups. As shown in Figure 2, group A exhibits distinct clustering compared to groups B and C. 

### 3.5. Microbial Community Composition

**Figure 3:**
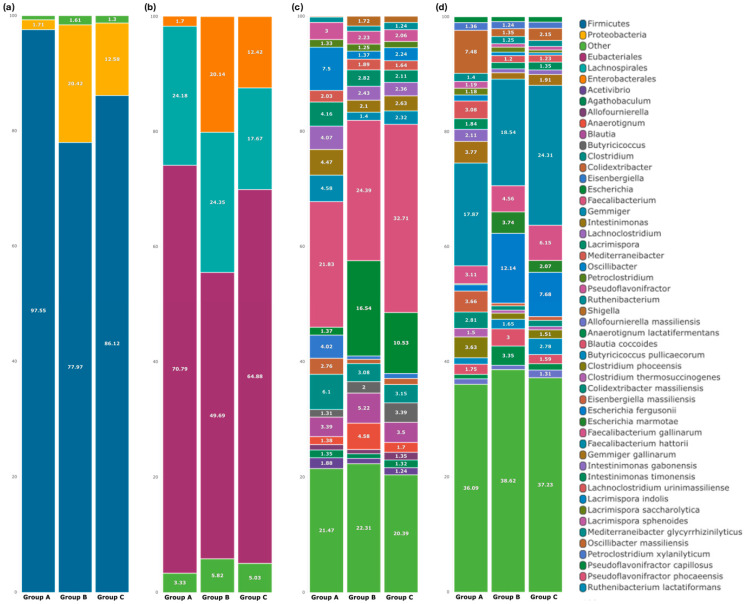
Taxonomic composition of cecal microbiota across experimental groups. Stacked bar plots represent the relative abundance (%) of bacterial taxa detected in cecal samples from group A, group B, and group C at four taxonomic levels; (**a**) phylum, (**b**) order, (**c**) genus, and (**d**) species. Only taxa with a relative abundance ≥ 1% in at least one group are shown individually; taxa below this threshold were grouped into the category “Other”. Taxonomic classification was based on Operational Taxonomic Unit (OTU) clustering and relative abundances were calculated as percentages of the total sequences per sample.

Most of the generated OTUs were assigned into 2 major phyla (90.9%), *Firmicutes* and *Proteobacteria*, 3 orders (90.9%), 21 genera (90.25%), and 25 species (90.2%) with a relative abundance greater than 1% (Figure 3; Table S4). Bacterial communities with relative abundances lower than 1% were collapsed into the “Other” category. As shown in Figure 3a, *Firmicutes* is the most dominant phylum across all groups with a relative abundance of 97.6% in group A, 78% in group B, and 86.1% in group C. Notable presence of Proteobacteria was also identified in all groups with a significant increase in group B (20.4%) compared to group A (1.7%). In group C, *Proteobacteria* relative abundance was 12.8%, lower than the challenged group. Phyla with relative abundance of less than 1% were clustered in the “Other” category (Figure 3a).

Order-level taxonomy classification is shown in Figure 3b. There are 3 orders identified in all groups, with *Eubacteriales* being the most abundant order in all of them (70.8% in group A, 49.7% in group B, and 64.9% in group C). The second most abundant order is *Lachnospirales*, with 24.2% relative abundance in group A, 24.4% in group B, and 17.7% in group C. *Enterobacterales*, which was notably increased in group B (20.1%) compared to the control group A (1.7%), showed a reduced relative abundance in group C (10.1%).

At the genus level, *Faecalibacterium* spp. is the most abundant in all groups, with a relative abundance of 22.3% in group A, 25.6% in group B, and 33% in the group C (Figure 3c). The second most abundant genus in group A is *Oscillibacter* spp. with a relative abundance of 7.7%. Although *Oscillibacter* spp. is also present in groups B and C, its relative abundance is lower than that in group A, with a percentage of 1.4% and 2.3%, respectively. *Clostridium* spp. is also present in all groups, with a relative abundance of 6.2% in group A and 3.2% in groups B and C. *Escherichia* spp. is the second most abundant genus in group B and group C, with a relative abundance of 17.4% and 10.6%, significantly higher than the relative abundance of *Escherichia* spp. in group A (1.4%). The notable presence of the genus *Blautia* spp. was observed in all groups, with 3.5% for control, 5.5% for the challenged group, and 3.5% for the vaccinated group. Lastly, *Shigella* spp. was also present in groups B and C in relative abundance of 1.8% and 1.1%, respectively, while in group A, *Shigella* spp. showed a relative abundance < 1%.

Classification of the observed OTUs in the species taxonomic level (Figure 3d) showed that the most abundant species in both groups is *Faecalibacterium hattorii*, with relative abundances of 17.9%, 19.5%s and 25.1% in groups A, Bs and C, respectively. The second most abundant species in group A is *Oscillibacter massilensis* (7.5%). Of note, *O. massilensis* abundance is lower in group B (1.4%) and group C (2.2%). In group B, the second most abundant species is *Escherichia fergusonii* (12.8%). *E. fergusonii* showed a relative abundance of 7.9% in group C, lower than that of group B. An increase in the relative abundance of *Faecalibacterium gallinarum* is also reported for group B (4.8%) and group C (6.4%), compared to group A (3.1%) (Table S4).

**Figure 4:**
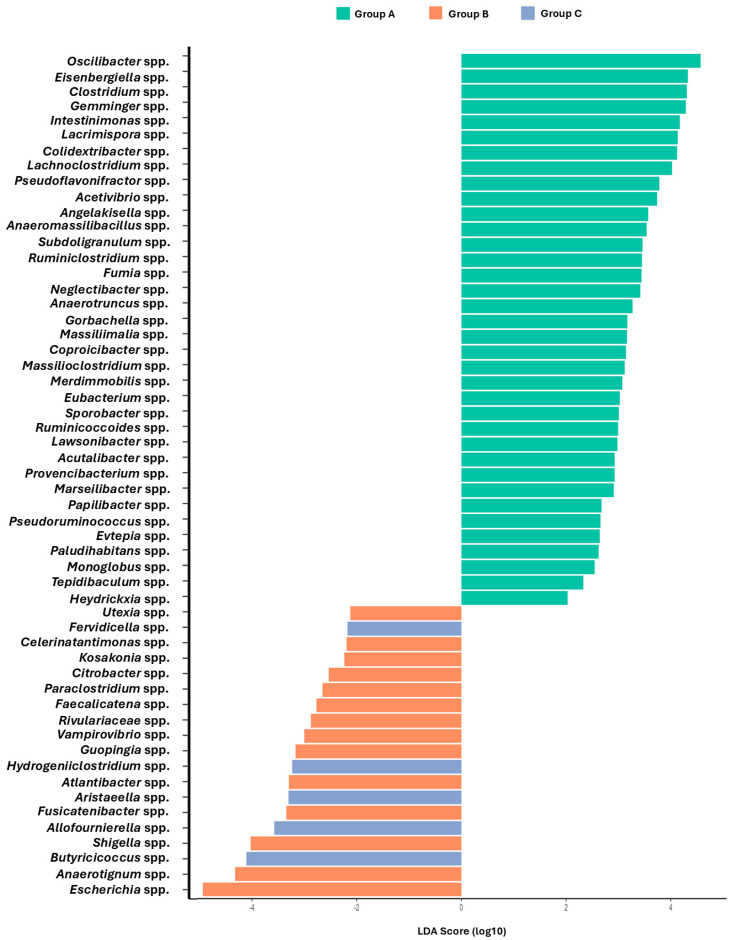
Differentially abundant bacterial genera among groups based on Linear discriminant analysis effect size (LEfSe). LEfSe was used to identify genera with significantly different relative abundances among group A (control), group B (challenged with *Eimeria* spp.), and group C (vaccinated and challenged with *Eimeria* spp.). Only genera with an adjusted *p* < 0.05 and LDA score (log_10_) above the significant threshold (log_10_ > 2) are shown. Genera enriched in group A are indicated by positive LDA scores (green), while genera enriched in group B and group C are shown with negative LDA scores in orange and blue, respectively.

To further explore differences in bacterial composition at the genus level, differential abundance analysis was performed. The analysis revealed statistically significant differences (0.01 < *p* < 0.05) in the relative abundance of bacterial taxa between the control group and the challenged groups B and C (Figure 4). Genera with positive LDA scores were more abundant in the control group, while those with negative LDA scores were more abundant in the challenged groups. *Oscillibacter* spp., *Eisenbergiella* spp., and *Gemmiger* spp. were among the genera with higher abundance in group A, along with *Clostridium* spp., *Intestinimonas* spp., and *Lachnoclostridium* spp. In contrast, group B was characterized by a higher relative abundance of *Escherichia* spp., *Anaerotignum* spp., and *Shigella* spp., with *Escherichia* spp. showing the strongest association. Group C exhibited an increased LDA score in genera such as *Butyrococcus* spp., *Allofournierella* spp., and *Aristeaeella* spp. compared to group A.

**Figure 5:**
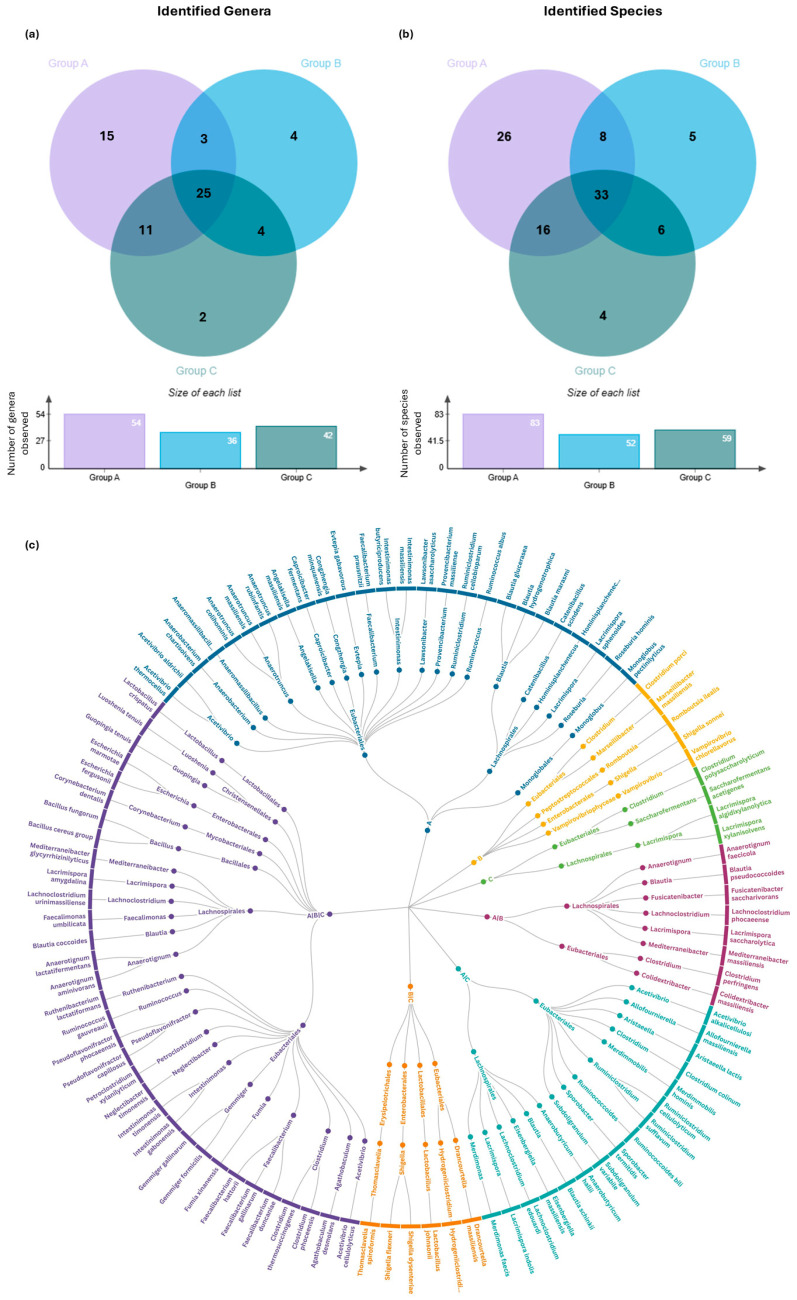
Comparative analysis of core microbiota composition across experimental groups. (**a,b**) Venn diagrams display the number of shared and unique bacterial taxa among group A (control), group B (challenged with *Eimeria* spp.), and group C (vaccinated and challenged with *Eimeria* spp.) at the genus (**a**) and species (**b**) level. Taxa were included only if they were detected in at least three replicates per group, representing the core microbiota per each condition. Bar plots below each Venn diagram indicate the total number of genera and species identified in each group, respectively. (**c**) Circular cladogram representing the taxonomic relationships and distribution of genus-level bacterial taxa identified across the groups. From the outer to the inner rings, the plot shows the following: species (outer ring), genera (intermediate ring), and phyla (central nodes). Each branch of the cladogram is color-coded according to group-specific or shared taxonomic presence; blue for taxa shared between group A, yellow for group B, green for group C, plum for taxa shared between groups A and B, turquoise for groups A and C, orange for groups B and C, and purple for genera shared among all three groups. The cladogram integrated taxonomic hierarchy with group distribution to highlight both phylogenetic relationships and taxa overlap across groups.

The distribution of microbial communities, at both the genus and species level, is illustrated in the Venn diagrams of Figure 5a,b. Genera present in at least 3 samples in each group were considered for the construction of the Venn diagrams. A total of 24 genera were shared between all groups, including *Faecalibacterium* spp., *Bacillus* spp., *Blautia* spp., and *Lactobacillus* spp. In group A, there were 15 genera that were not observed in either group B or group C, including members of *Evtepia* spp. and *Anaerobacterium* spp. Group B contained only 3 unique genera, *Colidextribacter* spp., *Faecalicatena* spp., and *Fusicatenibacter* spp., while group C had 2 unique genera, *Eshraghiella* spp. and *Saccharofermentans* spp. In terms of genera overlap, 11 genera were shared between groups A and C, including *Sporobacter* spp. and *Eisenbergiella* spp., but were not found in group B. Similarly, 4 genera, *Drancourtella* spp., *Hydrogeniiclostridium* spp., *Thomasclavelia* spp., and *Shigella* spp. were common between groups B and C (Table S5).

At the species level, 33 were shared among all three groups, 16 species were shared among groups A and C (such as *Acetivibrio alkalicellulosi* and *Sporobacter termitidis*), 6 between groups B and C (*Drancourtella massiliensis*, *Shigella dysenteriae*, and *Shigella flexneri*, etc.) and 8 between groups A and B (*Blautia pseudococcoides*, *Anaerotignum faecicola*, etc). Group A contained 26 unique species, including *Faecalibacterium prausnitzii*, group B had 5 species exclusive to it, including *Clostridium porci* and *Shigella sonnei*, and group C had only 4 unique species, like *Clostridium polysaccharolyticum* and *Lacrimispora xylanisolvens* (Table S5).

Genus-level overlap of bacterial communities among the groups is also visualized in a radial network graph, showing the linkage between genera and species alterations between groups (Figure 5c).

### 3.6. Predictive Functional Abundance Analysis

**Figure 6:**
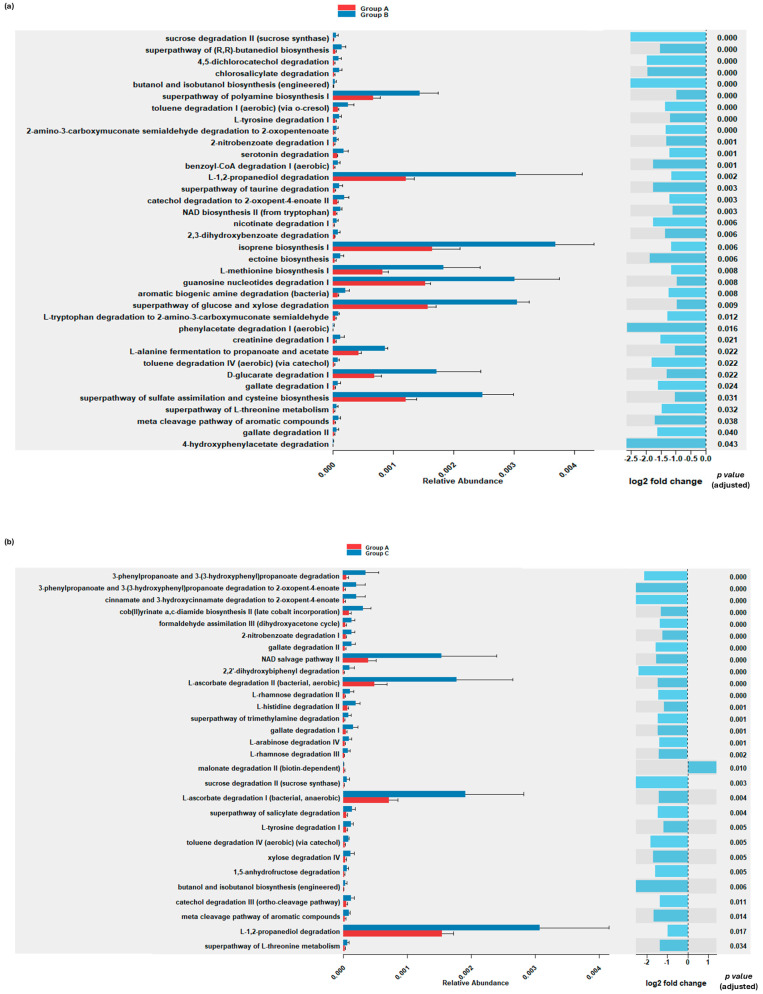
Predictive functional analysis of metabolic pathways differently enriched among experimental groups. (**a**) Relative abundance of key metabolic pathways in the control group (group A, red) and the challenged group (group B, blue), with the corresponding log2 fold change between groups displayed on the right. Pathways enriched in group A have positive log2 fold change values, whereas those enriched in group B have negative values. (**b**) Relative abundance of statistically significant metabolic pathways in the control group (group A, red) and the vaccinated and challenged group (group C, blue), with the corresponding log2 fold change displayed on the right. Pathways enriched in group A have positive values, while those enriched in group C have negative values. Statistical significance (adjusted *p*-value) for each pathway was calculated using the DESeq2 algorithm, embedded in ggpicrust2 R package, and is shown adjacent to the log2 fold change bars. Relative abundance of pathways with adjusted *p* < 0.05 were considered statistically significant.

To gain insight into the potential functional implications of the observed microbial shifts between groups, a predictive functional abundance analysis was performed, using picrust2 [54]. As shown in Figure 6a, group B showed a predicted enrichment in pathways related to carbohydrate metabolism (L-alanine fermentation to propanoate and acetate), amino acid metabolism (L-methionine biosynthesis I, L-alanine fermentation to propanoate and acetate and superpathway of polyamine biosynthesis I, superpathway of L-threonine metabolism, serotonin degradation, L-tyrosine degradatation I, etc.), lipid metabolism (isoprene biosynthesis I), sulfur metabolism (superpathway of sulfate assimilation and cysteine biosynthesis), and energy and fermentation pathways (L-1,2-propanediol degradation and L-alanine fermentation to propanoate and acetate) compared to group A (Table S6).

Concerning functional differences between group A and group C (Figure 6b, Table S6), enrichment in pathways related to amino acid and nucleotide metabolism (NAD salvage pathway II, L-tyrosine degradation, L-histidine degradation II, and superpathway of L-threonine metabolism) and carbohydrate metabolism (L-ascorbate degradation I, L-ascorbate degradation II, etc.) were predicted for group C. No significant difference in the predicted relative abundance of pathways between groups B and C was observed.

## 4. Discussion

Coccidiosis, caused by *Eimeria* spp., remains a major challenge for the poultry industry, impacting both animal welfare and sustainability [57]. While extensive research has explored its pathogenicity in broilers [58,59], the employment of high-throughput omics technologies [(meta)genomics, epigenomics, (meta)transcriptomics, (meta)proteomics, and metabolomics] can provide novel insight into *Eimeria* infection biology and support the design of efficient methods to combat this disease [15]. To that end, this study aimed to assess the performance, gross lesions, as well as structural and functional changes in the cecal microbiota of Ross 308^®^ broilers chicks following *Eimeria* spp. infection and anticoccidial vaccination.

In this study, gross lesions caused by *E. acervulina* and *E. tenella* were observed (7 dpi; 23 day) in the challenged group compared to the negative control group, highlighting the effectiveness of the infection protocol applied. Specifically, characteristic gross lesions of *E. acervulina* were observed and scored in the duodenum, consistent with the parasite’s known tropism for this anatomical part. Additionally, hemorrhages in the ceca were noted to be the result of *E. tenella* infection. However, birds that received vaccination prior to challenge exhibited significantly reduced lesion scores. This protective effect persisted through the second sampling (13 dpi; 29 day), where challenged birds continued to exhibit higher gross lesions than both the vaccinated and control groups. These findings suggest that vaccination mitigates the severity of *Eimeria* spp.-induced gut damage, likely by priming the immune response and reducing parasite replication [59,60].

The changes in intestinal gross lesions were accompanied by significant alterations in microbial diversity in the cecum. Alpha diversity indices revealed a marked decline in microbial richness and evenness in the challenge-only group compared to the control, indicating that *Eimeria* spp. infection disrupts microbiota stability, as indicated by Wu et al. [61]. Reduced microbial diversity is a hallmark of dysbiosis and may impair essential microbial functions, including nutrient breakdown, competition against pathogens, and immune regulation [7,40,62]. Interestingly, the vaccinated group exhibited numerically higher (non-significant), diversity levels compared to the challenged–unvaccinated group and significantly lower alpha diversity compared to the negative control group. A similar study by Cai et al. [23] also suggests a partial, non-significant preservation of microbial diversity after *Eimeria* spp. infection.

Beta diversity analysis further confirmed this disruption, as challenged birds exhibited distinct microbial community clustering, supporting the notion that infection induces substantial compositional shifts in the cecal microbiome [31]. The divergence in Bray–Curtis and Jaccard dissimilarity indices suggests that specific bacterial taxa are either depleted or enriched in response to infection [63]. However, 16S rDNA metagenomic studies by Chen et al. [34] and Su et al. [64] found no statistically significant microbial diversity between challenged and negative control groups, indicating that microbial alterations may depend on factors such as infection duration, chicken breed, and *Eimeria* spp. These discrepancies suggest that microbial alterations depend also on infection duration, chicken breed, and *Eimeria* spp. that caused the infection [34,65]. The vaccinated–challenged group also demonstrated distinct clustering compared to the control group and partial clustering to the challenged group, suggesting partial preservation of microbial composition but not complete prevention of dysbiosis, in agreement with Cai et al. [25].

The predicted upregulation of pathways associated with amino acid and polyamine metabolism, such as L-methionine biosynthesis I and L-alanine fermentation to propanoate and acetate, indicates that the microbial community responded to infection by increasing protein utilization [66,67,68]. Potential disruptions in amino acid balance may impact gut epithelium integrity, nutrient absorption, and immunity [69]. Additionally, the predicted enrichment of carbohydrate metabolism pathways, including L-1,2-propanediol degradation, could suggest a shift toward fermentation-based energy production, which may have altered nutrient absorption efficiency and further stimulated feed intake [70,71]. Predicted increased isoprene biosynthesis, associated with lipid metabolism, has been linked to oxidative stress and inflammation [61,71], further supporting that *Eimeria* spp. infection disrupts gut homeostasis [72].

ADWG was significantly lower (but biologically modest) in the unvaccinated–challenged group compared to the vaccinated–challenged group, which could be attributed to inefficient nutrient utilization due to infection-induced dysbiosis and metabolic shifts. The depletion of beneficial SCFA-producing genera, such as *Oscillibacter* spp. and *Eisenbergiella* spp., is particularly relevant, as SCFAs play a key role in gut health and energy metabolism [73,74]. Depletion of such beneficial genera was also observed by others [9,29,75]. Both *Oscillibacter* spp. and *Eisenbergiella* spp. are recognized as important SCFAs-producing bacteria in the gut microbiome. Reduction in challenged broiler chicks may suggest impaired SCFA synthesis, which is critical for maintaining epithelial barrier integrity, modulating inflammation, nutrient absorption, and growth performance. The increased abundance of *Enterobacteriales* spp., which includes opportunistic pathogens linked to gut inflammation [5], further supports the notion that infection compromised intestinal integrity, likely impairing nutrient absorption [66]. Additionally, the predicted alterations in lipid metabolism pathways, combined with the predicted increased carbohydrate fermentation, are consistent with the hypothesis that *Eimeria* spp. infection induces oxidative stress and inflammation [58], redirecting energy from growth toward immune function and gut repair [66].

Vaccination mitigated, but did not fully eliminate, the dysbiotic effect of coccidia challenge. The vaccinated group exhibited reduced levels of pathogenic genera, including *Escherichia* spp. and *Shigella* spp., compared to the challenged group, results that align with the findings of Macdonald et al. [76]. Some beneficial taxa, such as *Lactobacillales* spp. and *Clostridiales* spp., were more abundant in the vaccinated group than in the challenged group, though still lower than in the control group. These findings align with a study on Mahuang broilers vaccinated against coccidiosis, which showed microbial shifts towards a healthier composition with increased *Lactobacillus* spp. and *Bacteroides* spp. and reduced *Enterococcus* spp. levels, suggesting mitigation of inflammation-associated bacteria [25]. Regarding the predicted functional capacity of the gut microbiota of the vaccinated group, increased relative abundance of NAD salvage II and L-ascorbate degradation pathways was observed, potentially revealing a link between shifts in microbial metabolism and immune modulation and oxidative stress responses. NAD metabolism plays a key role in cellular energy homeostasis and immunity [77], while elevated L-ascorbate degradation may reflect microbial utilization of host-derived or dietary vitamin C, contributing to a more balanced microbiome [78,79]. However, full restoration of the gut microbiota structure and function was not observed, indicating that while vaccination offers protective effects, additional interventions, such as probiotics or dietary modifications, could further enhance gut health post-infection [59,80]. Finally, the absence of a vaccinated-only group (without *Eimeria* spp. challenge) limits our ability to assess the direct effects of vaccination on the gut microbiota. As a result, while our study provides insight into the combined effects of vaccination and challenge, we cannot conclusively determine the modulating properties of the vaccine alone.

Species-level analysis revealed that *Faecalibacterium hattorii* was dominant across all experimental groups. *F. hattorii* was recently described following the taxonomic reclassification of *F. prausnitzii* strains, with studies to date focusing primarily on its phenotypic and genomic features in human and mammalian hosts [81]. As a novel member of the *Faecalibacterium* genus, *F. hattorii*, like other species within this group, is believed to play a beneficial role in gut health, particularly through the production of short-chain fatty acids such as butyrate and its anti-inflammatory effects [82]. However, there are currently no studies confirming the presence or functional role of *F. hattorii* in broilers or poultry in general. Therefore, the detection of *F. hattorii* in our dataset provides novel insight and suggests that this species is present in the broiler gut. Further research using species-specific validation methods, such as shotgun metagenomics or targeted qPCR, is warranted to confirm its presence and assess its potential biological relevance in avian hosts.

It is important to highlight that in this study, microbiota analysis was conducted on pooled cecal content samples, which restricts our ability to assess individual-level variation in microbial composition and diversity. While pooling can reduce technical variability and cost, it may mask important host–microbiota interactions that occur at the individual level. In addition, microbiota profiling was limited to a single time point (day 23), which constrains our understanding of the dynamic changes in microbial communities throughout the course of infection, vaccination, and recovery. Future studies would benefit from longitudinal sampling to capture temporal trends and resilience of the microbiota.

A key limitation of this study, shared by most metagenomic analyses, is the inability to resolve microbial composition at the strain level. While our approach effectively identifies bacterial genera and species, distinguishing among specific strains remains a challenge due to inherent constraints in sequencing and bioinformatics methodologies. In particular, the Oxford Nanopore MinION platform, despite enabling long-read sequencing, is characterized by higher raw read error rates compared to short-read technologies [83]. However, real-time base-calling and implementation of better base-calling models can significantly increase the overall barcode quality [84]. Strain-level differences can significantly influence bacterial function, including probiotic properties, metabolic activity, and host interactions. In this context, new resources arise, including the Probio-ichnos database, to support the comprehensive cataloging of novel isolates and their phenotypic traits [85]. Furthermore, it should be stated that the functional metagenomic analysis is predictive, relying only on the full-length 16S rRNA gene sequencing results, and was not further validated. Future research, by integrating high-resolution metagenomic or culturomic approaches and metabolomics to assess the impact of microbial community alterations in the function of cecal microbiota, will assess strain-specific contributions to gut health and vaccine response.

Finally, although the experimental model aimed to reflect subclinical coccidiosis, as commonly recorded in commercial broiler production, it remains a controlled setting, and differences in management, stocking density, diet, and microbial exposure in commercial farms may influence both microbiota and disease dynamics [38,39]. Further in-field studies are required to determine potential breakpoint values and to assess the feasibility of using microbiome analysis as a non-invasive diagnostic tool for detecting subclinical coccidiosis without the need to sacrifice animals [86].

## 5. Conclusions

This study represents the first comprehensive analysis of both structural and functional aspects of cecal microbiota following *Eimeria* spp. infection using full-length 16S Oxford Nanopore sequencing combined with predictive functional metagenomics. The impact of multi-species *Eimeria* spp. infections on the cecal microbiota of broiler chicks was determined by integrating gross lesion scoring and metagenomics. The results indicate significant microbial dysbiosis in challenged birds, characterized by a reduction in beneficial SCFA-producing bacteria and an increase in opportunistic pathogens such as *Escherichia* spp. and *Shigella* spp. Gross lesion analysis confirmed intestinal damage, particularly in the ceca, aligning with microbial shifts observed in the challenged group. Predictive functional metagenomics revealed disruptions in metabolic pathways associated with amino acid metabolism, nucleotide degradation, and carbohydrate and lipid metabolism, suggesting potential implications for gut integrity, immune modulation, and nutrient absorption. Notably, vaccination partially mitigated dysbiosis but complete restoration of microbiota composition and function was not achieved. This study is limited by the implementation of predictive functional metagenomics, rather than direct functional assays or metabolomics, and by the use of Oxford Nanopore full-length 16S rRNA gene sequencing, which may reduce taxonomic resolution for closely related genera. Validating key microbial shifts and functional inferences using targeted qPCR and SCFA quantification would significantly strengthen future investigations. In addition, future research should focus on strategies such as probiotic supplementation, targeted nutrition, and advanced vaccination protocols to mitigate dysbiosis and improve poultry resilience against *Eimeria*-induced intestinal damage.

## Figures and Tables

**Table 1:** Effect of *Eimeria* spp. infection and its combination with anticoccidial vaccination on the performance parameters in broiler chicks ($\bar{x}$ ± SD).

Age/Period (days)	Group A (Negative Control)	Group B (*Eimeria* spp. Challenge)	Group C (*Eimeria* spp. Challenge and Vaccine)	*p*
	*Body weight (BW, g)*			
1st	44 ± 3	44 ± 3	45 ± 3	0.206
9th	165 ± 22	163 ± 22	158 ± 25	0.145
16th	416 ± 72	435 ± 52	425 ± 56	0.162
18th	544 ± 69	569 ± 63	553 ± 67	0.067
20th	681 ± 88	691 ± 77	680 ± 83	0.687
22nd	817 ± 102	812 ± 94	815 ± 93	0.954
24th	949 ± 126	943 ± 111	953 ± 117	0.895
29th	1383 ± 165	1364 ± 143	1390 ± 154	0.683
35th	1991 ± 232	1948 ± 210	2033 ± 222	0.265
	*Average daily weight gain (ADWG, g)*			
1–9	15.12 ± 1.42	14.82 ± 0.95	14.19 ± 1.01	0.383
9–22	50.18 ± 3.26	49.95 ± 1.31	50.58 ± 1.31	0.879
22–29	80.79 ± 3.46	78.98 ± 2.97	82.06 ± 5.00	0.412
29–35	101.44 ± 9.82	97.06 ± 7.14	107.28 ± 6.14	0.111
22–35	90.32 ± 4.53 ^a,b^	87.32 ± 3.62 ^a^	93.70 ± 2.60 ^b^	0.029
1–35	57.27 ± 1.64 ^a,b^	55.97 ± 1.51 ^a^	58.50 ± 1.04 ^b^	0.025
	*Average daily feed intake (ADFI, g)*			
1–9	14.42 ± 1.77	15.05 ± 1.78	14.58 ± 1.24	0.787
9–22	62.37 ± 1.99	61.70 ± 1.39	60.65 ± 1.07	0.181
22–29	104.16 ± 1.51 ^a^	106.65 ± 1.55 ^b^	107.74 ± 1.79 ^b^	0.005
29–35	132.58 ± 5.51	135.70 ± 8.80	132.30 ± 2.37	0.580
22–35	129.63 ± 3.86	131.92 ± 1.60	132.11 ± 2.25	0.247
1–35	73.41 ± 1.03	73.76 ± 1.06	73.39 ± 0.32	0.714
	*Feed conversion ratio (FCR g:g)*			
1–9	1.12 ± 0.08	1.19 ± 0.16	1.21 ± 0.12	0.427
9–22	1.34 ± 0.06	1.33 ± 0.03	1.29 ± 0.03	0.148
22–29	1.52 ± 0.05	1.60 ± 0.07	1.59 ± 0.08	0.150
29–35	1.66 ± 0.13	1.61 ± 0.09	1.55 ± 0.10	0.248
22–35	1.63 ± 0.06	1.67 ± 0.08	1.64 ± 0.09	0.856
1–35	1.49 ± 0.03	1.50 ± 0.03	1.47 ± 0.04	0.439

^a,b^ Means in the same row with a different superscript differ significantly (*p* ≤ 0.05).

**Table 2:** Effect of *Eimeria* spp. infection and its combination with anticoccidial vaccination on the coccidiosis lesion score (scale: 0–4) in the intestines of broiler chicks ($\bar{x}$ ± SD).

Day 23 (7 dpi)	Score	Group A (Negative Control)	Group B (*Eimeria* spp. Challenge)	Group C (*Eimeria* spp. Challenge and Vaccine)	*p*
		*Day 23 (7 dpi)*			
*E. acervulina*	0	18 (100%)	13 (72.2%)	17 (94.4%)	0.089
	1	0 (0%)	4 (22.2%)	1 (5.6%)	
	2	0 (0%)	1 (5.6%)	0 (0%)	
	3	0 (0%)	0 (0%)	0 (0%)	
	4	0 (0%)	0 (0%)	0 (0%)	
	AV	0.00 ± 0.00 ^a^	0.33 ± 0.59 ^b^	0.06 ± 0.24 ^a^	0.020
*E. tenella*	0	18 (100%)	0 (0%)	8 (44.4%)	
	1	0 (0%)	3 (16.7%)	7 (38.9%)	<0.001
	2	0 (0%)	15 (83.3%)	3 (16.7%)	
	3	0 (0%)	0 (0%)	0 (0%)	
	4	0 (0%)	0 (0%)	0 (0%)	
	AV	0.00 ± 0.00 ^a^	1.83 ± 0.38 ^c^	0.72 ± 0.75 ^b^	<0.001
	TMLS	0.00 ± 0.00 ^a^	2.17 ± 0.71 ^c^	0.78 ± 0.81 ^b^	<0.001
		*Day 29 (13 dpi)*			
*E. acervulina*	0	18 (100%)	16 (94.1%)	17 (94.4%)	0.585
	1	0 (0%)	1 (5.9%)	1 (5.6%)	
	2	0 (0%)	0 (0%)	0 (0%)	
	3	0 (0%)	0 (0%)	0 (0%)	
	4	0 (0%)	0 (0%)	0 (0%)	
	Total	18 (100%)	17 (100%)	18 (100%)	
	AV	0.00 ± 0.00	0.06 ± 0.24	0.06 ± 0.24	0.600
*E. tenella*	0	18 (100%)	4 (23.5%)	12(66.7%)	<0.001
	1	0 (0%)	10 (58.9%)	6 (33.3%)	
	2	0 (0%)	3 (17.6%)	0 (0%)	
	3	0 (0%)	0 (0%)	0 (0%)	
	4	0 (0%)	0 (0%)	0 (0%)	
	Total	18 (100%)	17 (100%)	18 (100%)	
	AV	0.00 ± 0.00 ^a^	0.94 ± 0.66 ^c^	0.33 ± 0.49 ^b^	<0.001
	TMLS	0.00 ± 0.00 ^a^	1.00 ± 0.61 ^c^	0.39 ± 0.61 ^b^	<0.001

^a–c^ Means in the same row with a different superscript differ significantly (*p* ≤ 0.05). Kruskal–Wallis test: day 23 (7 dpi): *E. acervulina*: group A vs. group B: *p* = 0.008, group B vs. group C: *p* = 0.034; *E. tenella*: group A vs. group C: *p* = 0.011, group A vs. groups B, C: *p* < 0.001; day 29 (13 dpi): *E. tenella*: group A vs. group B: *p* < 0.001, group A vs. group C: *p* = 0.054, group B vs. group C: *p* = 0.005. TMLS: total mean lesion score. AV: average.

**Table 3:** Effect of *Eimeria* spp. infection and its combination with anticoccidial vaccination on the dysbiosis lesion score (scale: 1–10) in the intestines of broiler chicks ($\bar{x}$ ± SD) on day 29 (13 dpi).

Dysbiosis Score	Group A (Negative Control)	Group B (*Eimeria* spp. Challenge)	Group C (*Eimeria* spp. Challenge and Vaccine)	*p*
1	0 (0%)	1 (5.6%)	0 (0%)	0.014
2	0 (0%)	0 (0%)	2 (11%)	
3	6 (33.4%)	0 (0%)	2 (11%)	
4	9 (50%)	11 (61.1%)	4 (22.2%)	
5	3 (16.6%)	4 (22.2%)	5 (27.8%)	
6	0 (0%)	2 (11.1%)	5 (27.8%)	
AV	2.83 ± 0.71	3.28 ± 1.07	3.50 ± 1.34	0.175

Kruskal–Wallis test: group A vs. groups B and C: *p* = 0.029. In this 0–10 dysbiosis lesion scoring, none of the intestines received a score of 7, 8, 9, or 10.

## Data Availability

The data presented in this study are freely available in the European Nucleotide Archive (ENA) at EMBL-EBI under accession number PRJEB87208.

## Supplementary Materials

The following supporting information can be downloaded at: https://www.mdpi.com/article/10.3390/microorganisms13071470/s1; Table S1: Ingredients and calculated analysis of the starter (1 to 10 days) and for finisher (10 to 35 d) diets; Table S2: Quality and yield characteristics of 16S DNA Sequencing Reads; Table S3: Microbial alpha diversity per Sample; Table S4: Relative abundance percentages; Table S5: Venn diagram of taxonomic distribution; Table S6: Differential abundance analysis of MetaCyc metabolic pathways between groups.
