# Supplementary material for: Metagenomic Insight into Cecal Microbiota Shifts in Broiler Chicks Following Eimeria spp. Vaccination
Source: Microorganisms. 2025 Jun 24;13(7):1470. doi: 10.3390/microorganisms13071470 (PMC12300358; doi:10.3390/microorganisms13071470)
Supplement: Supplementary file 1 [file microorganisms-13-01470-s001.zip › Table S1.pdf]

Article

Metagenomic insight into cecal microbiota shifts in broiler chicks following *Eimeria* spp. vaccination

Dimitrios Marinos Karadedos, Tilemachos Mantzios, Despoina Eugenia Kiousi, Margaritis Tsifintaris, Ilias Giannenas, Panagiotis Sakkas, Georgios A. Papadopoulos, Gunther Antonissen, Aglaia Pappa, Alex Galanis and Vasilios Tsiouris

Table S1. Ingredients and calculated analysis of the starter (1 to 10days) and for finisher (10 to 35 d) diets.

Ingredients (g/kg feed)	Starter (1-10 days)	Finisher (10-35 days)
Wheat	461.5	390
Soybean meal (CP 47%)	304	273
Corn	100	212
Soybean oil	50	55
Fish meal 65%	25	12.3
Nutriphos 22%	15.3	12.7
Calcium carbonate	12.8	10.9
Phytogenic fat (99%)	-	20.5
Gluten 60%	14.2	-
Adisodium	2.4	2
Threonine-L	1.3	0.5
Choline CL 50%	0.2	-
Methionine DL	2.1	1.7
Lysine HCL	2.7	1.5
Salt	1.5	1.9
Vitamin premix2	2	2
Nutrients premix3	2	2
Clinacox	0.2	0.2
Smectagri	2	2
Xylanase 8000 G	0.3	0.3
Calculated analysis (g/kg)		
Moisture	10.48	10.51
Dry matter	89.41	89.38
Crude protein	22.84	19.50
Crude fibre	2.34	2.29
Crude fat	6.74	9.45
Crude Ash	6.25	5.45
Starch	35.50	38.00
Methionine	0.54	0.46
Methionine + Cysteine	0.99	0.84
Lysine	1.41	1.15
Threonine	0.96	0.77
Tryptophan	0.28	0.24
Arginine	1.47	1.29
Valine	1.06	0.92
Leucine	1.76	1.51
Isoleucine	0.96	0.83
Linoleic acid	3.34	5.75
Calcium	0.97	0.79

Phosphorus	0.78	0.66
Sodium Chloride	0.15	0.19
Energy (Kcal/kg)	3018.00	3200.26

[2]Contents per kg: 6,000,000 I.U. retinyl acetate; 2,000,000 I.U. cholecalciferol; 40,000 mg DL-α-tocopheryl acetate; 4,500 mg menadione sodium bisulphite; 1,500 mg thiamin; 3,500 mg riboflavin; 3,000 mg pyridoxine HCl; 12.5 μg cobalamin; 25,000 mg niacin; 75 mg biotin; 750 mg folic acid; 7,500 mg pantothenic acid.

[3]Contents per kg: 200,000 mg choline chloride; 25,000 mg Fe; 45,000 mg Zn; 65,000 mg Mn; 10,000 mg Cu; 750 mg I; 150 mg Se.
